# Assessing progress in managing and improving quality in nascent integrated care systems in England

**DOI:** 10.1177/13558196231209940

**Published:** 2023-11-01

**Authors:** Mirza Lalani, Priya Sugavanam, James Caiels, Helen Crocker, Sarah Gunn, Harriet Hay, Helen Hogan, Bethan Page, Michele Peters, Ray Fitzpatrick

**Affiliations:** 1 Department of Health Services Research and Policy, 4906 London School of Hygiene and Tropical Medicine, London, UK; 2 Nuffield Department of Population Health, 6396 University of Oxford, Oxford, UK; 3 Personal Social Service Research Unit, 2240 University of Kent, Canterbury, UK; 4 113089 Picker Institute Europe, Oxford, UK; 5 Department of Experimental Psychology, 6396 University of Oxford, Oxford, UK

**Keywords:** integrated care, quality of care, quality management

## Abstract

**Objectives:**

In 2022, England embarked on an ambitious reorganisation to produce an integrated health and care system, intended also to maximise population health. The newly created integrated care systems (ICSs) aim to improve quality of care, by achieving the best outcomes for individuals and populations through the provision of evidence-based services. An emerging approach for managing quality in organisations is the Quality Management System (QMS) framework. Using the framework, this study assessed how ICSs are managing and improving quality.

**Methods:**

Four ICSs were purposively sampled, with the data collected between November 2021 and May 2022. Semi-structured interviews with system leaders (*n*=60) from health and social care, public health and local representatives were held. We also observed key ICS meetings and reviewed relevant documents. A thematic framework approach based on the QMS framework was used to analyse the data.

**Results:**

The ICSs placed an emphasis on population health, reducing inequity and improving access. This represents a shift in focus from the traditional clinical approach to quality. There were tensions between quality assurance and improvement, with concerns that a narrow focus on assurance would impede ICSs from addressing broader quality issues, such as tackling inequalities and unwarranted variation in care and outcomes. Partnerships, a key enabler for integration, was seen as integral to achieving improvements in quality. Overall, the ICSs expressed concerns that any progress made in quality development and in improving population health would be tempered by unprecedented system pressures.

**Conclusion:**

It is unclear whether ICSs can achieve their ambition. As they move away from an assurance-dominated model of quality to one that emphasises openness, learning and improvement, they must simultaneously build the digital infrastructure, staff expertise and culture to support such a shift.

## Introduction

Health and social care systems in England are facing the unprecedented pressures of increasing needs from an ageing population, rising workload for an overburdened workforce and limited financial resources.^1^ There is a growing consensus that better integration of care is a key part of the approach to tackling these challenges.^2^ ‘Integration’ is used interchangeably but represents a ‘joining up’ of traditional silos of care across (horizontal) and within (vertical) systems, organisations, services and service providers.^3^ To address these multiple challenges England embarked on an ambitious national re-organisation in 2022, designed to produce a unified and integrated health and care system, intended to maximise population health. Similar ambitions are being pursued by many high-income countries so the potential to learn lessons from the English experiment is substantial.^4^

Since the introduction of the 2012 Health and Social Care Act in England, there have been several efforts to integrate care: ‘Vanguard’ sites to test ‘New Care Models’,^5^ Sustainability and Transformation Plans and Accountable Care Organisations.^6^ Each of these developments was underpinned by a premise of transferring care away from hospitals to community settings, as well as increased collaboration between individual institutions complemented by a place-based population health focus.^7^ The 2019 National Health Service (NHS) Long Term Plan included the aim that the entire country be covered by around 40 integrated care systems (ICSs). These were expected to bring health and care organisations together to work more effectively on a broad population-level agenda, including prevention, addressing health inequalities, improving care outcomes and better management of resources. Legislation enabling the creation of the ICSs was implemented in July 2022.^8^

### Governance of ICSs

The newly developed ICSs in England include Integrated Care Boards (ICBs), which replace Clinical Commissioning Groups (CCGs) in NHS planning functions. ICBs’ leadership teams comprise a range of senior leaders from across health, social and voluntary care, with the added responsibility of involving local people and communities in strategic planning and governance.^9^ ICSs also include Integrated Care Partnerships, which operate as statutory committees and comprise senior representatives of NHS organisations and local government (known as local authorities in England), as equal partners with a wider focus on public health and social care. Their primary responsibility is to develop a strategy, outlining how the ICS can deliver its goals, with the ICBs being part of the delivery mechanism. Both Integrated Care Partnerships and ICBs are expected to interact with NHS and local government at place level, ensuring local level strategies are being considered in planning and commissioning.^10^

### Managing quality in an integrated care system

Quality can be described as the degree to which services for individuals and populations increase the likelihood of achieving desired health outcomes.^11^ The National Quality Board’s (NQB) guidance for ICSs in England considers quality to be a multi-dimensional concept with the following characteristics: ‘Safe … Effective … [a] Positive experience ... Well-led … Sustainably-resourced … equitable’.^12(p3)^ Using this framing, ICSs will be responsible for achieving the best outcomes for individuals and populations through the provision of evidence-based services, whilst avoiding harm, and promoting a positive care experience. Evidence of variability in health and clinical outcomes across the country highlights the need for quality improvement at the system level.^13^ ICSs’ management of quality will be critical to addressing these disparities.

There are well-established approaches to manage and improve quality in health care organisations, solving problems using specific methods and tools, with the aim of fostering measurable improvement within a health care setting.^14^ However, there are fewer whole-systems approaches to managing quality with a limited understanding of system-wide progress, and few documented initiatives that have made positive contributions to improving quality at scale.^15^ One such approach is the Quality Management System (QMS), which can be used by organisations as a practical tool to help organise quality-related activities. The framework aims to enable the delivery of good quality care. Extending beyond quality improvement to include other key components of quality – planning, control and assurance – which together form a holistic approach to quality at all levels of an organisation.^16^

A key challenge for QMS is to balance activity, resource and efforts across the four domains: planning as a regular activity; improvement used intermittently to enhance performance; assurance to check whether standards are maintained; and control to manage daily performance as a team, service or Board.^17^ Bringing together these elements of quality across a whole system, not just an organisation, will be a significant endeavour for nascent and still evolving ICSs. To date, QMS has been applied to a small number of individual hospitals or Trusts. We will use the framework to support our understanding of how quality might be addressed at the level of a whole health and care system.

This study uses the QMS framework to assess how ICSs are managing and improving quality. The study will also provide insights into the progress ICSs have made in terms of integration, using the development of quality as a lens.

## Methods

We used a qualitative case-study approach, comprising semi-structured interviews, meeting observations and documentary review. The research was conducted prior to the ICSs gaining a statutory footing, that is, while ICSs were still evolving, and the pace and scale of development of the quality system varied across the four ICSs. Nonetheless, the NQB and NHS England (NHSE) have produced guidance on their expectations for ICSs in developing quality. Hence, our initial discussions indicated that ICSs were converging toward similar structures and governance for quality.

### Study setting

We purposively sampled four ICSs, ensuring variation in terms of geography (urban/rural), demography (population size), and pre-existing system architecture (e.g. length of history as an ICS) or related experiences (e.g. had Vanguard status). Details are in Table 1. Constituent organisations in each ICS included acute Trusts, community and mental health Trusts, GP practices, ambulance Trusts, local authorities and Healthwatch (a place-based organisation that collects patient and user feedback on their experiences of using health and social care services).

**Table 1.** Key characteristics of the four ICSs in this study.

ICS	Characteristics
A	Population 1 million. Four place-based partnerships covering rural and urban areas. Formed as an ICS in 2019 (3 years prior to official launch). Some history of partnership working across health and care at the system level.
B	Population 1.8 million. Five place-based partnerships covering several rural and urban areas. One of the places was awarded Vanguard status (support and funding to develop innovative models of care which other parts of the country can learn from). Limited history of partnership working at system level.
C	Population 1.1 million. Five place-based partnerships covering a large and primarily rural area with one major urban centre. Limited history of partnership working at system level.
D	Population 2 million. Seven place-based partnerships in a major urban centre. One of the places was awarded Vanguard status. Formed as a Sustainability and Transformation Partnership prior to becoming an ICS, and comprised three sub-system partnerships. Extensive history of partnership working across health and care at sub-system and system level.

### Data collection and analysis

Interviews, observations of meetings and the collection and analysis of relevant documentation took place between November 2021 and May 2022. An overview of the data-collection activities is provided in Table 2.

**Table 2.** Data-collection activities.

Research method	Data sources
Interviews (*n*=60)	ICS executive team: chief executive, independent chair, chief information officer, medical director, other executive members. Senior representatives in NHS Acute and Community Mental Health Teams, CCGs: • Chief executive/deputy chief executive • Chief nurse • Director of nursing • CCG clinical chair • CCG directors: transformation, performance, assurance • Quality leads: chief nurse, chief quality officer, lead for quality development, clinical quality manager Local authority representatives: Director of adult and children services, director of public health Healthwatch, Voluntary Community and Social Enterprise, and other public involvement representatives
Observations of meetings	Meetings included: • Integrated Care Partnership Board • Integrated Care Board • CCG Governing Board • Quality forum • System Quality Group • System Delivery Group • ICS executive team
Documentary analysis	Several documents pertaining to ICS and quality, including: ICS strategic plans, ICS quality strategy and framework

Semi-structured interviews (*n*=60) were held with senior leaders and other key stakeholders across the four ICSs. Interviews were guided by a topic guide, focussing on overall ICS perspectives on quality, how the ICS is organised to address quality, the internal and external influences on the ICS’s approach to quality, challenges faced and the capacity of the ICS to address quality, including the role of data. All interviews were conducted on Microsoft Teams and lasted between 45 and 70 min. We also attended several relevant meetings at the system level, which provided context for the ongoing work on integration and quality within the ICS. Documents from each of the ICSs were also reviewed, which supported our understanding of quality structures, governance and strategies.

Interviews were audio-recorded and transcribed verbatim. We conducted qualitative analysis using a thematic framework approach to identify patterns and themes in the data.^18^ The qualitative data management tool NVivo v12.0 was used to manage and code the interview data.

The QMS framework was used to organise, categorise and facilitate interpretation of the data. Some elements of the QMS were adapted to ensure it aligned with a systems approach to quality management in the context of ICSs. The research team met at frequent intervals during data collection and analysis to discuss identified themes and to develop a coding framework which was updated iteratively. The analysis was informed by the literature on quality in care and ICSs as well as organisational literature on managing change, complex decision-making processes and implementation.

### Ethical approval

Ethics approval was provided by the University of Kent (ref LSSJ0459). Researchers approached interview participants by email outlining the purpose of the study and the interview process. Written informed consent was obtained from each participant prior to interview. Participants were assured of confidentiality and anonymity and that participation was voluntary, and that they were free to withdraw from the study. No participants withdrew their consent.

## Results

The study findings are arranged as per the four domains of a QMS: planning, control, assurance and improvement. We also give results for a fifth theme: partnership – that is, working with system partners and local people and communities to underpin quality development and enable integration.

Of the four participating ICSs, one had formally adopted the QMS approach to support quality development, and all expressed an interest in exploring its use further. For an overview of the quality approaches employed by the ICS to develop their quality systems, see the online supplement.

### Quality planning

Quality planning aims to understand the needs/assets of service users and residents – identifying gaps and risks in the system and to use this information to set priorities for improvement and design a strategy to meet these priorities.

#### Strategy and priority setting

Several interviewees mentioned that the overall strategy for their ICS aimed to address the population’s needs. System priorities also influenced quality goal setting, although this was largely underdeveloped at the time of our study. Strategies and priorities differed across the four ICSs. One determined that their quality strategy should be completely aligned to the overarching ICS strategy, as quality was perceived as the crucial mechanism for ensuring the delivery of the ICS’ goals of reducing inequalities and tackling variation in access, experience, and outcomes. Instead of a separate quality strategy, they created a Quality Framework which reiterated the centrality of the key ICS priority areas and a focus on developing an open improvement culture, transparency in decision-making and a QMS approach as key pillars for delivering ICS goals. Another ICS placed population health centred on the life course and the reduction of inequalities at the heart of their approach to quality, thus aspiring to have an impact on the overall health of the population rather than simply service improvement.

It was acknowledged that quality was one of many competing priorities for the ICBs, with the risk of certain priorities such as prevention being derailed by day-to-day operational risks such as ambulance handovers and longer-term system pressures such as elective backlogs exacerbated by COVID-19. There was also some debate regarding the balance between national, system and local place-based priorities:

Because the major problem system leaders are facing at the moment is dealing with the fallout from the pandemic, rather than actually concentrating on quality of service, we’re getting a lot of feedback about the waiting lists. Patients, you know, have a horrendous waiting list due to the pandemic, so sometimes I think they’re going to have bigger priorities to worry about. (Healthwatch representative)

There were signs of collaboration across system partners, including with local authorities and Healthwatch in identifying quality priorities. But, overall, the quality agenda was dominated by health care partners. The system-orientated strategic approach to quality, reflected in the quality goals, signalled a greater emphasis on access and equity, although it was unclear how these intentions would be operationalised:

The population health approach is essentially trying to pull the themes from public health into an NHS lens so that it’s about looking at whole populations, not just the patient in front of somebody. It involves trying to get good outcomes for populations and to try to reduce inequalities and be more preventative, so it’s that more holistic view of how health and wellbeing is promoted in populations through the delivery of health and social care services. (Public health director)

#### Establishing structures and roles for quality

In establishing structures and roles for quality we observed the sheer scale and complexity of arrangements largely imposed centrally. The legacy of CCGs remains intact with their previous responsibilities for quality assurance and risk management retained by ICBs. Moreover, while the System Quality Group comprises a diverse membership, leadership for quality development is held by the NHS through the Chief Nurse, with some concerns that this may result in a narrow health care-centred perspective for quality.

### Quality control

This involves establishing performance standards and near real time measurement systems that highlight areas for intervention.

#### Identifying measures and metrics

Many interviewees suggested that multiple health-orientated indicators exist for service and provider-level assurance, but there remained a relative lack of timely data that could highlight variation in performance and guide the focus of intervention. Data was also lacking from primary and social care, preventing an overview of quality across care pathways, as well as data and metrics on the success of integration. Links between health care data and local authority data were also weak:

It’s a major issue for us. What we’ve got is literally hundreds of old bits of software by departments that don’t really talk to each other, aren’t bolted together properly and are underpinned by reams of paper files. I’ve still got a warehouse with seventeen miles of patient paper records. It constrains my ability to undertake good research. It definitely has an impact on quality metrics. (Chief nurse)

ICSs were in the process of identifying outcome measures and metrics that aligned with their system priorities, but were mindful of not placing undue burden in terms of data collection. One ICS was trialling a selection of measures across performance, quality and transformation for each place.

There was an appetite to move to more outcome-based measurement and the development of person-centred outcomes. This signalled a shift of focus from tracking individual provider/service performance and quality to an expanded person/population-centred focus.

#### Analytics capacity

Individual organisations were considered to have varying levels of analytical capacity and there was recognition of the need to invest in building this capacity. More generally, it was felt that ICSs needed to look at how organisations could work together strategically to share existing capacity, resources and data.

#### Data collection and analysis

All systems were working towards integrating data sources across population-level data, secondary care, primary care, social care and voluntary services. Integrated care records, which collect data as people move across several services, offered the possibility of real-time data collection. But at the time of our study, these were still evolving and did not cover the whole system. Adding insights from local people and communities, was thought essential, supplementing hard data with a diverse range of perspectives and narratives allowing clearer focus as to the needs of local communities:

In terms of the quantitative data, we’ve got a lot of it and it’s useful if we know how to understand it and use it to inform what we do. But it’s only half the story … we really need to look at how are we looking at things in a qualitative way and that’s where it comes back to the patient experience stuff, the patient stories and bringing that in. (Director of nursing)

Adequate funding and partnering with universities, research teams or Healthwatch were mentioned as enablers to facilitating better data collection and analysis. The Chief Information Officer in each ICS hoped for a future in which fully integrated partner records, real-time systems with sophisticated analysis and feedback to support quality improvement, and the use of mobile technology to transform care provision were commonplace. This vision was tempered by the reality of limited national progress in developing dynamic information systems and limited resources locally.

### Quality assurance

Quality assurance is focussed on internal and external mechanisms for oversight of performance, conformity to standards and identifying gaps and risks.

#### External guidance and standards

Interviewees mentioned an emerging system model for assessment and regulation – the Care Quality Commission’s single assessment framework.^19^ Two of the participating ICSs had contributed insights and/or acted as pilot sites for this initiative. One of these ICSs perceived the new assessment framework as an opportunity to prioritise engagement of local authorities in both system-level strategic decision-making for quality as well as improvement work at place.

There was some uncertainty about the boundaries for assurance and regulation between NHSE, the Care Quality Commission and ICSs in the fxd future. Concerns were expressed that the system’s efforts to take a proactive response to tackling longstanding complex system quality issues might be subsumed by a much larger role of assurance of partnerships and individual organisations. Moreover, there was a broad consensus that ICSs were feeling overburdened by multiple requests for data/information from different parts of NHSE, both regionally and nationally, to fulfil assurance requirements. These concerns illustrated an underlying tension between assurance and improvement. This was compounded by the reportedly top-down approach to quality imposed by arms-length bodies that focussed on performance management, targets and metrics primarily applied to NHS organisations, particularly acute Trusts. This approach was in contrast to the nationally set goals for ICSs of improving population health and reducing inequalities:

Although NHS England is the instigator, basically, of the whole creation of ICSs, when you listen to xxxx and the team talking, they’re talking about electives, they're talking about vaccination, they’re talking pandemic, they’re talking about, you know, long waiters. They are not talking about prevention, wider determinants of health, the local authority involvement, social care, everything that underpins the ICS. (ICS executive team member)

#### Internal oversight

It was thought that ICSs would focus on assurance to meet the demands of national bodies. This preference would be compounded by the retention of CCG staff and their historical focus on performance. Some considered this to be a parochial approach, with the ICSs paying greater attention to areas of risk and safety rather than tackling wider issues such as inequalities:

It’s the burning issues that seem to be getting attention – those we’re not doing so well on. That’s why assurance will predominate. Particularly while the people that are currently managing the quality system in the ICS are people who have a commissioning background, and, so, are very assurance-based in their thinking … It’s going to be difficult to change the thinking around quality to something more modern and innovative. (Quality lead)

Local authorities considered the assurance-focussed approach as an inhibiting factor for integration. They felt the NHS was preoccupied by governance and bureaucracy, which impeded progress on collaborative working.

### Quality improvement

Quality improvement is a structured approach to system redesign based on known improvement methods.

#### Resources for quality improvement

Several interviewees alluded to concerns regarding adequate resources for quality improvement – in terms of training, skills and expertise – and the requisite amount of personnel. It was thought that existential pressures on the health and care system would see resources devoted primarily to assurance requirements, leaving less available for improvement work. Even so, each of the ICSs was committed to enhancing their quality improvement capacity and capability through establishing ‘quality academies.’ These are hubs, whose purpose is to provide improvement-focussed training and a platform for sharing relevant knowledge, skills and expertise for the benefit of all system partners.

#### Leadership for quality

Strong leadership was thought to be required to deliver a quality-system that balanced assurance with learning and improvement. Such leadership would facilitate alignment of local and system priorities, help build trust and promote transparency encouraging the sharing of problems and promote a learning culture such that improvement becomes everyone’s business. In some ICSs, leadership for quality had expanded beyond the NHS through the establishment of a Chief Quality Officer role. It was anticipated that this development might promote wider engagement and have more salience with local authorities.

There were concerns about a lack of quality improvement capability at all levels of the system, but particularly among system leaders. These limits, exacerbated by an ingrained mindset focussed on a risk-averse model of quality based on assurance, were seen as a significant barrier to the necessary paradigm shift required to make progress on improvement:

Who is going to lead this work? … They’re not going to have expertise in it sitting around the ICS table if they are recruiting to clinical leads for medicine, or medical and nursing, and finance. So where are they going to get the expertise to be able to design and deliver this kind of work? (Quality lead)

For some ICSs, drawing on the expertise of provider organisations within the system that already had mature programmes for quality improvement was key to implementing system-wide quality improvement.

#### Maximising the use of data

Data was seen as integral to identifying areas for quality improvement and tracking progress. As previously mentioned, significant barriers hindered the utility of data to support quality in ICSs. This was due to variable progress on identifying meaningful measures, data sharing across the system, analytic capacity and the presentation and timeliness of information.

### Partnerships

#### Partnerships with system and place partners

System leaders acknowledged the value of broad partnerships for making progress on quality. The COVID-19 response had played a key role in accelerating partnerships across systems, by bringing health, social care services and community providers closer together. Historical partnerships at sub-system level had built a foundation for this to happen. There were caveats though, as some local authorities and Healthwatch considered themselves not to be equal partners. The barriers to establishing effective partnerships included health-centred and technocratic language and framing, as well as the NHS’s proclivity for bureaucratic governance.

#### Partnerships with local people and communities

There was broad acknowledgment of the significance of local people and communities in redesigning services to address unmet need and reduce unwarranted variation. Partnerships with local people and communities were mentioned as being critical for building trust to share problems and find solutions.

Voluntary organisations and local authorities were seen as sources of expertise in engagement. These organisations, in turn, felt that ICSs needed to develop a more inclusive and tailored approach to public involvement. However, some leaders felt that there was an over-reliance on Healthwatch as the voice of people using services. They felt there was a risk this dependence led to a limited view of issues and that more diverse voices representative of local communities were not being heard:

I would like patients and users to be heard at every stage of everything we do. And the challenge I always get is, ‘But we need the right people.’ And the default always seems to be Healthwatch. I don’t think that’s right, because they’re not selected, they’re kind of – you know, sometimes they come en masse and they’ve all got a different approach but they’re there to represent. And you think there needs to be some guidelines and boundaries about how we do that. (ICS executive member)

## Discussion

The reorganisation of health and care in England away from a competition-based model of commissioning and providing services to the provision of an integrated and whole-population service is an ambitious step that warrants close attention, given similar aspirations internationally. This study provides some novel insights into the early stages of development of ICSs, using quality as a lens. Our use of the QMS framework has provided a greater understanding of quality development, identifying the key functions required to manage and improve quality in ICSs. Moreover, the study findings indicate that partnership working, a well-established enabler for integration^20^ is also a key facilitator for quality development.

The findings suggest that data, measurement and metrics are important considerations for ICSs. Optimising use of data to derive meaningful metrics of quality – which enable close to real-time recognition of unwarranted variation in performance and timely intervention – is critical, if systems are to have any chance of achieving their goals. Multiple bodies from national executive agencies to think tanks offer ‘key metrics’ for judging progress, for example, the Institute of Public Policy Research has developed an outcome-based integration index that reflects performance across organisational boundaries.^21^ It is challenging for ICSs to judge the value or validity of these proposed new measures, which remain to be tested in real-world contexts. Whether this work needs to take place nationally or at system-level, there is a need for rationalisation of the number of current measures and identification of those that are most likely to drive change and improvement.

Current performance measures, such as non-elective admissions or length of stay, are limited in terms of measuring quality in integrated systems.^22^ National measures can lack timeliness and fail to provide local contextual information. Given these limits, there is scope for ICSs to develop their own user-friendly presentations and broaden the scope of quality measures to include key underlying drivers, such as staff and patient satisfaction, and to incorporate a population health perspective. Encouragingly, the recently launched Care Quality Commission’s single assessment framework sets out an intention to base assessment on a broader array of quality of care outcomes across safety, experience, equity and access, while also examining progress on the relational features of integration, such as partnership working between providers and with local people and communities.^19^ Progress will be slow without improved integration of care records and data sources that shed light across whole care pathways and open up the opportunity for continuous data collection and analysis.

Additional organisational constructs may be needed to understand how ICSs address responsibilities, such as quality. For example, Hammond and colleagues developed the concept of ‘meta-governance’ to understand the increasing role of arms-length bodies to mediate between central government policy and service delivery.^7^ The effect of such third parties is to develop and oversee implementation of policies in highly complex areas, such as health and care, in lieu of government. It may be that ICSs are emerging to play such an important role but may end up competing with NHSE as a developer and implementer of policy.

Similarly, Denis and colleagues develop the concept of collective leadership as a commonly proposed solution in the field of health.^23^ A key point they make is that such arrangements can be fragile if role ambiguities are high. Again, such a perspective underlines potential risks of ambiguity between ICSs and NHSE, which is evident in this study through the tensions we have identified between quality assurance and improvement. These tensions have been exacerbated by the centralised and top-down approach of arms-length bodies, which are parochially focussed on performance management and assurance to the possible detriment of tackling the broad scope of quality issues they face (including addressing inequalities and unwarranted variation in care and outcomes). Rebalancing quality approaches to incorporate a wider role for quality improvement is not straightforward for several reasons. Firstly, national guidance places emphasis on assurance and the management of risk as a key focus for quality leaders at system level. Secondly, the legacy of CCG ways of working make an assurance-orientated approach to quality a more natural fit, both for staff and in terms of the types of data collected. Thirdly, despite an evidence base that is voluminous, clarity with regards to the best approach for quality improvement is limited.^24^ Indeed, improvement initiatives may not only have positive but also negative consequences and, hence, alone, are not necessarily the solution for service improvement in complex care systems.^25^ A systems approach to quality improvement could be strengthened by considering measures of staff wellbeing, team relationships and culture. Such measures would allow consideration of the most impactful areas to focus on to achieve improvement – be that the environment within which staff work, enacting policies that promote a just culture and encouraging partnership working. Recent work provides helpful clues as to how initiatives to promote quality improvement at a system level could develop: for example, taking more account of the role of regulators,^26^ improvement approaches applied to population health,^27^ and the need for more evaluative rigour in selection of improvement methods.^28^

Assessing progress on integrated care was not the main purpose of the study, but it was difficult to demarcate challenges associated with integration from those of quality development. The study was undertaken at time when ICSs were still forming, with several organisations across the ICSs having seldom worked together. While the development of ICSs in England was the natural endpoint of the Lansley reforms of 2012, their passing into legislation is set against the backdrop of the COVID-19 pandemic and its additive effect on already struggling health and care systems. The evidence for integration measured in terms of health service outcomes remains largely mixed and benefits are rarely witnessed in the short term.^22^ Furthermore, any progress in terms of quality that extends beyond the traditional dimensions to address equity, access and inequalities are likely to stall given the unprecedented pressures on the care system.

We used the QMS framework to organise our data and to support our understanding of quality development in the context of an ICS, adapting the Shah et al.^17^ version of the framework to apply at the system level. Our findings reveal that the QMS framework is a useful in guiding systems in organising their quality related activities, but has limited scope to act as an explanatory tool to assess the impact and effectiveness of quality development approaches. To enhance its practical function, its scope could be extended to include dimensions such as relationships/partnerships and leadership which are important enablers for quality improvement.^29^

Quality Improvement collaborations have proven effective in certain clinical areas.^30^ However, they require time and staff expertise, which may be in short supply given current pressures to produce immediate results. Softer processes, such as establishing partnerships, are key to creating a context that supports improvement across the system, but take great effort given historical organisational divisions. Greater involvement of local authorities and voluntary organisations in strategic decision making for quality as well as at operational levels is imperative to successfully tackling system-level quality problems and maintaining momentum in improving population health and reducing inequalities.

There was an emerging aspiration across ICSs in this study to eventually become learning systems, using real-time integrated data to drive decision making and identify areas of risk at an early stage. However, this ambition remains a distant prospect due to existing information governance barriers, complex data sharing agreements and fragmented data systems across health and social care. Policymakers must ensure that data is an enabler for integration and quality development, not a barrier. This includes taking account of the potential workload placed on systems in responding to data requests. There are some useful examples of good practice, including integrated care records, but further benefit could be garnered from whole systems datasets which would incorporate local authority data.^31^ Moreover, as a priority, there is need to identify and utilise measures of integration that are informative to systems in terms of directing where to intervene.

The wide range of factors impacting on population health is substantial and complex, including individual behavioural risk factors, socio-economic factors (such as income and employment status), and environmental circumstances (such as housing and the physical environment). Whilst the evidence-base for the need to address such factors is robust, the evidence for effective interventions to reduce inequalities is more complex.^32^ We struggled to identify how ICS are planning to achieve this goal, particularly at the system level. Much of the work on reducing inequalities will be undertaken at place level, but the roles of place and system will need further specifying if lines of accountability for outcomes are to be agreed and duplication avoided. As part of efforts to reduce inequalities, ICSs have adopted the national Core20PLUS5 programme.^33^ This presents an opportunity to shift the mindset of NHS organisations, who have traditionally seldom considered inequality reduction within their remit. Nonetheless, the programme’s clinically oriented approach, centred on secondary prevention, risks side-lining local authorities, whose Public Health Directorates possess expertise on inequalities reduction, retain a unique insight into the needs of their local populations (including unmet need) and focus primarily on primary prevention.

### Limitations

There was one main limitation to our study. All interviewee participants were senior system leaders and, hence, may have provided an ‘elite account’, expressing opinions and perspectives on behalf of their organisation rather than their personal views.^17^ To mitigate this risk we built relationships with quality leads in each of the ICSs, who informed other colleagues of our research well in advance.

## Conclusions

The formal launch of ICSs heralded a major change to the delivery of health and social care in England, with a scale of ambition not seen before. ICSs now have responsibility for managing the health of the wider population residing within their boundaries. This represents a seismic shift in policy. There are huge pressures on health and care systems to focus on key operational issues, such as reducing the post COVID-19 backlog, and managing urgent and emergency care. These pressures draw attention and resources away from managing quality. ICSs clearly face challenges both within all quality domains and in sustaining efforts to improve population health and address inequalities.
